# Dose-related adaptive reconstruction of DMN in isoflurane administration: a study in the rat

**DOI:** 10.1186/s12871-023-02153-6

**Published:** 2023-06-28

**Authors:** Fengru Guo, Yuqin Li, Zhaoxin Jian, Yan Cui, Wenhui Gong, Airui Li, Wei Jing, Peng Xu, Ke Chen, Daqing Guo, Dezhong Yao, Yang Xia

**Affiliations:** 1 Department of Neurosurgery, MOE Key Lab for Neuroinformation, Sichuan Provincial People’s Hospital, University of Electronic Science and Technology of China, Chengdu, 611731 China; 2 Department of Physiology, School of Basic Medicine and Tongji Medical College, Huazhong University of Science and Technology, Wuhan, 4030030 China

**Keywords:** Isoflurane, Default mode network

## Abstract

**Background:**

The anesthetic states are accompanied by functional alterations. However, the dose-related adaptive alterations in the higher-order network under anesthesia, e. g. default mode network (DMN), are poorly revealed.

**Methods:**

We implanted electrodes in brain regions of the rat DMN to acquire local field potentials to investigate the perturbations produced by anesthesia. Relative power spectral density, static functional connectivity (FC), fuzzy entropy of dynamic FC, and topological features were computed from the data.

**Results:**

The results showed that adaptive reconstruction was induced by isoflurane, exhibiting reduced static and stable long-range FC, and altered topological features. These reconstruction patterns were in a dose-related fashion.

**Conclusion:**

These results might impart insights into the neural network mechanisms underlying anesthesia and suggest the potential of monitoring the depth of anesthesia based on the parameters of DMN.

**Supplementary Information:**

The online version contains supplementary material available at 10.1186/s12871-023-02153-6.

## Introduction

General anesthesia (GA) is a reversible unconsciousness state induced by anesthetics. This state is characterized by amnesia, analgesia, and akinesia while maintaining physiological stability during surgeries [1]. However, inadequate administration of anesthetics may result in intraoperative awareness, leading to negative psychological effects and potential medicolegal issues [2, 3]. Conversely, excessive dosages of anesthetics can cause physical harm or even death [4, 5]. It is crucial to carefully monitor and adjust anesthesia levels to ensure patient safety and successful surgery outcomes.

Despite the long history and extensive exploration of the GA, there is still a lack of reliable methods for anesthetic release and anesthesia monitoring [6], which is partly due to the ongoing mystery surrounding its mechanism of action. Current research efforts are aimed at uncovering this mystery by studying molecular target mechanisms, and brain regions based on target neural pathways of general anesthetics [7]. However, there is limited research on the interactions among brain networks in a dose-related fashion, which could serve as a promising avenue for exploring GA-induced unconsciousness [8].

Clinical and laboratory research involving resting-state functional magnetic resonance imaging (fMRI) studies the effect of anesthesia on the nervous system, indicating that large-scale resting-state networks (RSNs) show functional disconnection and network segregation under anesthesia [9–11]. These temporal-spatial alterations illustrate that information integration within RSNs is disrupted under GA, which hints at the potential mechanism of the loss of consciousness [12]. Whereas, some other studies showed that functions subserved by several higher-order brain networks, such as the default mode network (DMN), are retained until higher doses of anesthetic are administered [13], and the increment of the functional connectivity in the isoflurane state can be found [14], and functional connectivity (FC) in the DMN covaries with anesthetic dose [15]. Based on these results, the effects of isoflurane dose on the brian network were still unclear. While, the fMRI based on the blood oxygenation level-dependent signal has a too low temporal resolution to reflect more detailed aspects of neuronal activity. Fortunately, electrophysiological signals have the potential to remedy this limitation.

In addition, the DMN as an evolutionarily conserved RSN acts as a set of brain regions that persist in relatively synchronous activity in the quiet-awake (QA) resting state [16–18], which has been presumed to be associated with self-referential functions [19–21]. As the DMN exists in humans, nonhuman primates, and rodents [19], rodents act as a great preclinical model to research the function of the DMN under GA. Prior studies have suggested that DMN performed highly correlated with GA [15]. However, there were rare studies to explore the alteration of DMN from QA to anesthesia states induced by different anesthetic doses systematically. Hence, we hypothesize that the electrophysiological DMN was reconstructed and covaried with anesthetic doses, adaptively. We implanted electrodes in the rats' DMN regions illustrated in Lu’s article [16] to record local field potentials (LFPs) in QA and anesthesia states with the administration of a stepwise increase of isoflurane doses.

Herein, isoflurane is one of the most widely used anesthetics in preclinical research. The levels of isoflurane in this article were adjusted from 0.75%, which minimally immobilizes the animals, and 1.25%, which is deep enough for surgery, but burst-suppression activity has not yet appeared, to 1.75%, which was deep enough to induce burst-suppression pattern.

## Materials and methods

### Animal preparation and LFP data acquisition process

Twenty adult male Sprague–Dawley rats (300-350g; Chengdu Dossy Experimental Animals Co., Ltd) were utilized following the National Institutes of Health guidelines, and experimental protocols were approved by the Ethical Committee on Animal Experimentation of the University of Electronic Science and Technology of China (UESTC).

Animals were housed five per cage in temperature-controlled IVC cages (Suzhou Fengshi Laboratory Equipment Co., Ltd) with food and water ad libitum before surgery. Animals were implanted with chronic homemade electrodes (insulated nichrome probes for eight deep brain regions, diameter: 200 m$\mu$, resistance: 70–700 $k\Omega$; stainless-steel screws for eight cortical brain regions, diameter: 500 m$\mu$, resistance: 2–40 $k\Omega$) in DMN areas reported in an fMRI study [16] and a reference electrode (stainless-steel screw; diameter: 500 $\mu m$, resistance: 2–40 $k\Omega$) in the cerebellum. During surgery, the animals were anesthetized with 1.5% isoflurane (4% for induction and 1.5–2% for maintenance, RWD Life Science) in the air (R540 anesthesia machine, RWD Life Science), positioned in a stereotaxic frame (68,028, RWD Life Science) using blunt ear bars, and maintained at a body temperature of 37 °C (RWD-69020, RWD Life Science). All stereotactic coordinates for each electrode were relative to the bregma and are shown in Table 1. After implantation, all the electrodes were assembled into the connectors and cemented to the skull of the rat with dental acrylic. After the surgery, all animals were fed in a clean cage while individually housed to recover for at least two weeks before local field potential (LFP) recording.

**Table 1:** Coordinates of the 15 electrodes

Region	Paxino’s atlas		
	AP (mm)	ML (mm)	DV (mm)
PrL	4.2	± 0.8	3
OFC	3.7	± 1.8	4.7
CG	1.7	± 0.7	2.6
RSC	-3.3	0	0
Hip	-4.3	± 1.4	3
PPC	-4.5	± 4	0
V2	-5.2	± 2.4	0
TeA	-5.2	± 8	5

*PrL* Prelimbic cortex, *OFC* Orbitofrontal cortex, *Cg* Cingulate cortex, *RSC* Retrosplenial cortex, *Hip* Hippocampus, *PPC* Posterior parietal cortex, *V2* secondary visual cortex, *TeA* auditory/temporal association cortex

After recovery from surgery, each rat was habituated for at least 3–5 days before the experiments. For baseline data acquisition, LFPs in the free-moving state were obtained for 30 min. Thereafter, before anesthetic data recording, the animals were rapidly anesthetized with 4% isoflurane, air flow rate (0.8 L/min), and moved into a stereotaxic frame, and then isoflurane was discontinued. When animals’ senses and movement were restored, isoflurane was immediately switched to 0.75%. Animals’ body temperature was controlled and maintained. Three levels of isoflurane doses were tested, including 0.75%, 1.25%, and 1.75% isoflurane, in each animal, and spontaneous LFPs were monitored for 10 min after an equilibration time of 20 min during each dose, the data recording procedure and original data were partly shown in S1. The LFP signals were obtained by a Symtop amplifier device (UEA-FZ 41; Symtop Instrument Co., Ltd, Beijing, China) and custom-made LFP recording software. The signals were sampled at 1000 Hz and bandpass filtered for 0.1–30 Hz with a notch filter to reject the 50 Hz line noise.

Respiratory rate (RR), the response to forepaw stimuli (RFS), and movement status (M) was also recorded before LFP data recording in each condition. And burst-suppression ratio (BSR) was calculated according to LFP (detailed information and RR, RFS, M, and BSR were shown in *Supplementary materials*).

After the examination, the animals were sacrificed, and performing histology examination for further analysis. Detailed information could be found in *Supplementary materials*.

### LFP data analyses

#### Data selection and preprocessing

This study included 600s LFP datasets under three isoflurane doses and selected 300 s QA LFP datasets without noise and movement artifacts for each animal. First, to remove low-frequency drift and high-frequency noise, the data were bandpass filtered (second-order Butterworth filter using zero-phase forward and reverse algorithm) within a frequency range of 1–30 Hz for each LFP segment. Second, these datasets were bandpass filtered into delta (1–4 Hz), theta (4–8 Hz), alpha (8–13 Hz), and beta (13–30 Hz) bands. Finally, we segmented the filtered data into 5 s segments in length for subsequent analysis.

The raw datasets were processed offline by using the functions in the MATLAB (R2020b; MathWorks, Inc. USA) toolbox EEGLAB (version 14.1.0; EEGLAB (ucsd.edu)[22] and custom MATLAB scripts.

#### Power spectral density

**Fig. 1:**
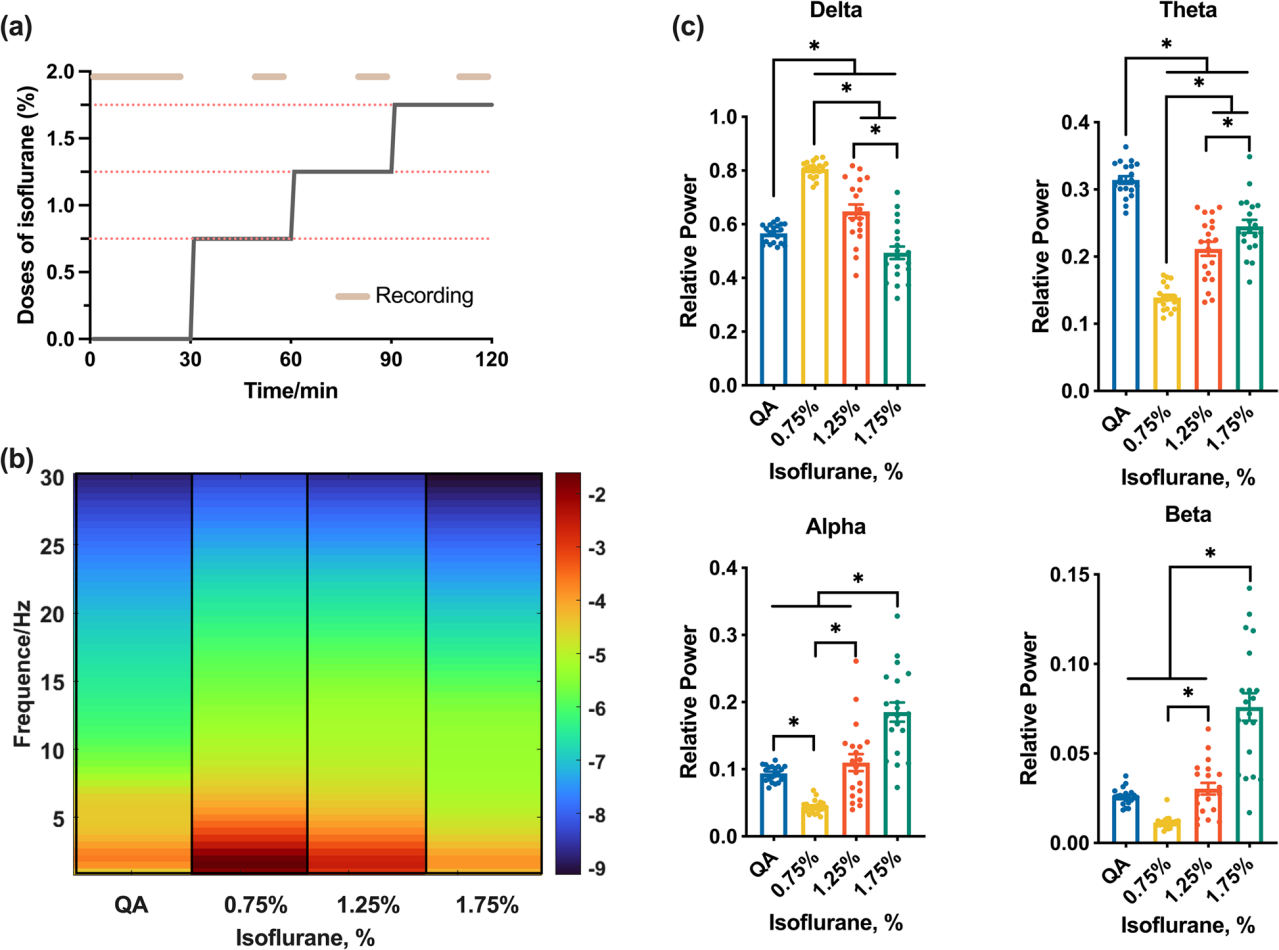
The procedure of isoflurane administration and LFP PSD during different states. **a** Schematic procedure of the experiment. The isoflurane was administered at sustained concentration and increased in a stepwise fashion. **b** Average LFP spectrogram during four states from 20 animals. The color bar indicates the log PSD. The QA and four isoflurane-dose states were demarcated by the black box in each spectrogram. **c** Relative power spectrum density in delta, theta, alpha, and beta band. Dark blue columns refer to the QA state, yellow columns refer to the 0.75% isoflurane, and red columns refer to the 1.25% isoflurane, green columns refer to the 1.75% isoflurane. QA, Quiet awake state. The dots refer to the relative power values of each rat. One-way ANOVA with ﻿Bonferroni’s post hoc test. ∗ indicates a significant difference between states (QA, 0.75%, 1.25%, or 1.75%) with *p* < 0.05; *n* = 20

The critical feature of the isoflurane effect on the nervous system is oscillation alteration, especially the low-frequency activity. To estimate the transformation of the LFP activity under isoflurane manipulation, the power spectrum density (PSD) based on the Fourier transform was utilized. For Welch’s method, the 5s epoch was divided into 2s sub-epoch with 90% overlap, and the power spectra of each sub-epoch were calculated using a Hamming window (MATLAB functions ‘pwelch’). The average spectrogram of DMN was estimated by averaging brain regions and rats (Fig. 1b).

The relative power (RP) of each frequency band was calculated based on pre-resulted PSD. The absolute power (AP) spectra of DMN in each frequency range per animal should be the result first. The AP spectra of DMN were calculated by averaging all the resulting power spectra of each sub-epoch per animal. The RP spectra of each animal were calculated by dividing the AP spectra in each frequency range by the total AP (1–30 Hz).

#### Functional network

To better understand the alterations anesthesia imposes on the DMN, we estimated the FC and built the FC topological network of DMN regions, via calculating the phase-lock values (PLVs) (MATLAB, R2020b). The PLVs algorithm was used for estimating the phasic relationship of two signals, which has been reported as a reliable method of neuronal communications among brain regions [23–25]. First, the selected LFP signals of QA and three doses of isoflurane were bandpass filtered in delta, theta, alpha, and beta frequency bands. Second, with the Hilbert transform, pairwise PLV matrices were built between every electrode pair, which were ranging from 0 to 1 to reflect the strength of the synchronous activity between two LFP signals.

For the ‘static’ functional estimation, the corresponding adjacency matrix with dimensions of 15 $\times$ 15 was then obtained for each artifact-free 5 s-length segment and then constructed the topological network. Furthermore, the final functional networks for the four states were constructed by further averaging the adjacency matrices across all 5 s-length segments [detailed information is given in the article [26]. The overall functional connection strength (FCS) of each rat was obtained by averaging brain regions in 15 $\times$ 15 matrices.

#### Fuzzy entropy of network

Brain functions fluctuated not only during high vigilance states but in unconsciousness states. This dynamic organization of the resting-state activity could reflect the special spatiotemporal variation imposed by stepwise isoflurane on the brain network [27]. To evaluate the dynamic properties, a ‘time-series’ functional connectivity estimation was conducted using fuzzy entropy. Fuzzy entropy is sensitive to the transient-related information content of signals polluted by noise [28, 29]. First, the preprocessed segments were divided into 10 s sub-epochs that overlapped 99% for computing PLVs to obtain $N$ networks of each rat. Corresponding series of each network connection can be defined as ${Y}_{k}\left(1\le k\le N\right)$ formulated as follows:

$$\begin{array}{c}{Y}_{k}^{p}=\left\{v\left(k\right), v\left(k+1\right),...,v\left(k+p-1\right)\right\}-\overline{v }\left(k\right),k=1,\dots ,N-p+1\left(1\right)\end{array}$$

where ${Y}_{k}^{p}$ indicates $p$ continuous $v$ values (i.e. PLV value) at the $k-$ th network point, which is generalized by removing the baseline $\overline{v }\left(k\right)={p}^{-1}{\sum }_{l=0}^{p-1}n\left(k+l\right)$.

After that, the similarity index ${D}_{kl}^{p}$ was obtained between two adjacent vectors, ${Y}_{k}^{p}$ and its ${Y}_{l}^{p}$, which is defined as:

$$\begin{array}{c}{D}_{kl}^{p}=\mu \left({d}_{kl}^{p},r\right)\left(2\right)\end{array}$$

Here, ${d}_{kl}^{p}$ indicates the maximum absolute difference in the scalar components between ${Y}_{k}^{p}$ and ${Y}_{l}^{p}$.

For each vector ${Y}_{k}^{p}\left(k=1, 2,..., N-p+1\right)$, corresponding ${\varphi }_{k}^{p}\left(r\right)$ was then calculated as follows:

$$\begin{array}{c}{\varphi }_{k}^{p}\left(r\right)={\left(N-p-1\right)}^{-1}{\sum }_{l=1, l\ne k}^{N-p}{D}_{kl}^{p}\left(3\right)\end{array}$$

The $FuzzEn\left(p,r\right)$ of the time series ${Y}_{k}\left(1\le k\le N\right)$ was defined as follows:

$$\begin{array}{c}FuzzEn\left(p,r\right)=\underset{N\to \infty }{\mathit{lim}}\left[ln{\varphi }^{p}\left(r\right)-ln{\varphi }^{p+1}\left(r\right)\right]\left(4\right)\end{array}$$

Which can be estimated by the statistic:

$$\begin{array}{c}FuzzEn\left(p,r,N\right)=ln{\varphi }^{p}\left(r\right)-ln{\varphi }^{p+1}\left(r\right)\left(5\right)\end{array}$$

Here, $p$ and $r$ indicate the embedded dimension and similarity boundary, respectively. As proved, $r$ was set as 0.2 and $p$ set as 2 [30, 31].

Thereafter, the top 20% variability of network edges with the largest fuzzy entropy of sub-epochs was used to identify the flexibility of the network architectures of the DMN, and the lowest 20% variability was used to index the stability of the network [32]. 20% smallest and largest fluctuation FCS were resulted by averaging 20% smallest and largest fluctuation FC, respectively.

#### Topological features of the DMN

Thereafter, multiple network properties, such as modularity (*Mod*) and clustering coefficients (*Cluster*) were further considered as a quantitative measurement of the inherent significant differences among these states. The *Mod* is commonly used to index the integrative capacity of a network, and the *Cluster* reflects the capacity to maintain functional specialization in the network [8, 33]. All the fully-connected weighted adjacency matrices were without threshold strategy for following properties of a static network computed. The following are detailed definitions by which *Cluster* and *od* were computed in this study.

For *Cluster,* the strong *Clusters* of FC indicated the strong communication within DMN regions. The ${PLV}_{ij}$ represented the FC between nodes $i$ and $j$, and ${L}_{ij}$ is denoted as the shortest path length between two nodes, $N$ represents the number of all notes, and $\Theta$ represents the set of network nodes. Then the *Cluster* was computed as follows:

$$\begin{array}{c}Cluster=\frac{1}{N}\sum_{i\in \Theta }\frac{\sum_{j,l\in \Theta }{\left({PLV}_{ij}{PLV}_{il}{PLV}_{jl}\right)}^{^{1}\!\left/ \!_{3}\right.}}{\sum_{j\in \Theta }{PLV}_{ij}\left(\sum_{j\in \Theta }{PLV}_{IJ}-1\right)}\left(6\right)\end{array}$$

The modularity capacity of the network is referred to by $Q$ [33, 34], which tends to be high if the brain network exhibits high functional segregation. The score $Q$ was defined as follows:

$$\begin{array}{c}Q=\frac{1}{2m}\sum_{ij}\left({A}_{ij}-\gamma \frac{{k}_{i}{k}_{j}}{2m}\right)\delta \left({c}_{i},{c}_{j}\right),\left(7\right)\end{array}$$

Here, the ${A}_{ij}$ represented the weight of FC (PLV) between DMN nodes $i$ and $j$, $m=\frac{1}{2}{\sum }_{ij}{A}_{ij}$. ${k}_{i}={\sum }_{i}{A}_{ij}$ as the total FC across all connections with nodes $i$, ${c}_{i}$ is the community to the assigned nodes $i$. $\gamma$ is the structural resolution free parameter (set to 1) and $\frac{{k}_{i}{k}_{j}}{2m}$ is the expected null FC defined with ${k}_{i}={\sum }_{i}{A}_{ij}$. $\delta \left({c}_{i},{c}_{j}\right)$ is the Kronecker $\delta$ function and equals 1 if nodes $i$ and $j$ belong to the same community and 0 otherwise.

These topological properties were calculated by using the Brain Connectivity Toolbox [35] (MATLAB, R2020b).

#### Statistical analysis

Prism 9.0 (GraphPad) was used for statistical analysis and to graph data. Statistical To determine the significance of differences in the vital parameters in the DMN during the four states (QA, 0.75%, 1.25%, and 1.75% isoflurane), Brown-Forsythe and Welch ANOVA tests with Dunnett’s T3 post hoc tests were applied, and *p* < 0.05 was set as the significance level.

For PSD and network properties, potential differences in related networks among the four states were first statistically investigated by using one-way repeated analysis of variance (ANOVA) with Bonferroni post hoc tests. We used the simple linear-modeling regression to estimate the relationship with isoflurane doses and the parameters resulted in previous (FCS, 20% smallest fluctuation FCS, 20% largest fluctuation FCS, PSD, *Mod*, *Cluster*).

## Results

### PSD of four frequency bands under different isoflurane doses

We analyzed the inherent oscillatory properties of the DMN associated with QA and three-step increased isoflurane-dose states, e.g., 0.75%, 1.25%, and 1.75% isoflurane states (Fig. 1a). The spectrogram of DMN showed distinct changes as the rats received increasing doses of isoflurane. The LFP activity in the four frequency bands was increased during the lowest dose of isoflurane state, while the activity was pull-back with the increment of doses (Fig. 1b).

To find out the relative variation of each frequency band, we measured the relative power spectral densities (RP) of LFP in four frequency bands (delta: 1–4 Hz; theta: 4–8 Hz; alpha:8–13 Hz; beta: 13–30 Hz). The results of RP within DMN suggested that the variation tendency of the delta band was different from the other three higher-frequency bands. More specifically, the delta band activity was intensive under the lowest dose of isoflurane state (Fig. 1c, delta: QA vs. 0.75%,* p* < 0.01, one-way ANOVA with Bonferroni’s post hoc test, *n* = 20), but with the doses of isoflurane gain it was decreased (Fig. 1c, delta: 0.75% vs. 1.25% and 1.25% vs. 1.75%: *p* < 0.01, one-way ANOVA with Bonferroni’s post hoc test, *n* = 20). For theta, alpha, and beta frequency bands, the RP of DMN was decreased in 0.75% isoflurane-dose state, which seemed that the activity of the DMN was mainly in the delta frequency band. With the isoflurane gaining, alpha and beta activity were revered to be intense (alpha: QA vs. 0.75%, beta: QA vs. 0.75%: *p* < 0.01, one-way ANOVA with Bonferroni’s post hoc test, *n* = 20). On the contrary, RR, RFS, M, and BSR of the rats under different isoflurane doses showed that the vital sign, capability of response, and nervous system was suppressed by isoflurane (Stable 2).

### DMN FC under different isoflurane doses

**Fig. 2:**
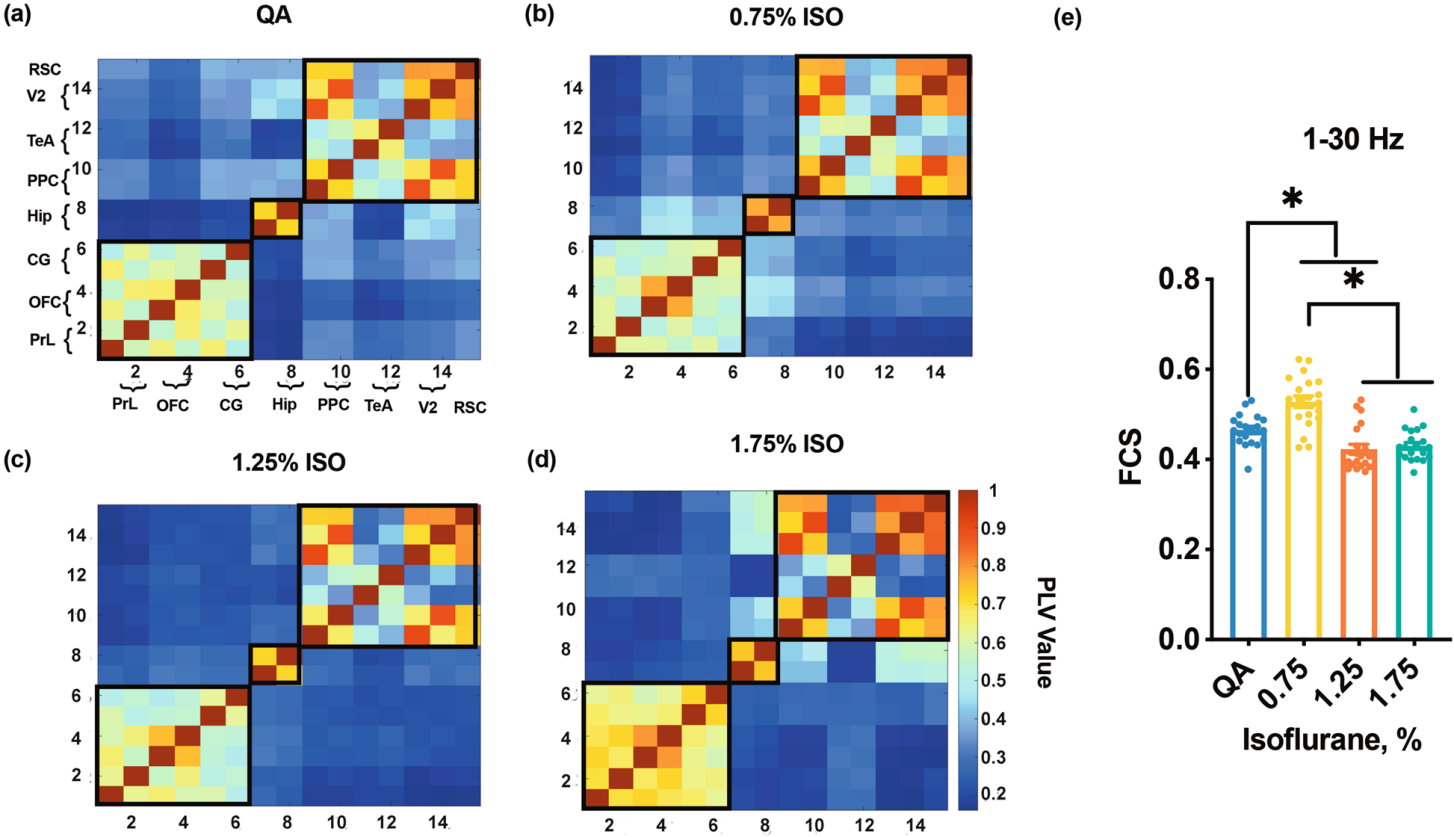
Functional connectivity under QA and three isoflurane-dose states in the 1–30 Hz. **a**-**d** The topology of DMN under QA and three isoflurane-dose states in the 1–30 Hz. QA, Quiet awake state. PrL, prelimbic cortex; OFC, orbital cortex; Cg, cingulate cortex; Hip, hippocampus; PPC, posterior parietal cortex; TeA, auditory/temporal association cortex; V2, secondary visual cortex; RSC, retrosplenial cortex. **e** The Holistic FCS of the DMN. FCS, functional connection strength. The dots refer to the FCS of each rat. ∗ indicates a significant difference between each state (QA, 0.75% isoflurane, 1.25% isoflurane, and 1.75% isoflurane) using one-way ANOVA with a Bonferroni post hoc test. The level of significance is *p* < 0.05. *n* = 20

We evaluated the topologic structure in 1–30 Hz and individual frequency bands (delta, theta, alpha, beta) to explore FC among DMN regions under different isoflurane-dose states. For this analysis, the DMN was a modularized organization (displayed in black boxes in Fig. 2), and the strong FC was found in the anterior part (prelimbic cortex (PrL), orbitofrontal cortex (OFC), and cingulate cortex (CG)), bilateral hippocampus (Hip), and the posterior part (secondary visual cortex (V2), temporal association cortex (TeA), and retrosplenial cortex (RSC)), which were common existed in the QA and three isoflurane-dose states in all frequency bands (Fig. 2a-d, S3). Under different isoflurane-dose states, DMN topological structure was adaptively reconstructed and displayed more modularized and segregated, especially in the posterior part of the DMN (Fig. 2b-d, S3).

Additionally, Fig. 2e shows the overall FCS of the DMN, which suggested that the FCS was increased under the lowest dose of isoflurane (0.75% vs QA: *p* < 0.01. One-way ANOVA with Bonferroni's post hoc test, *n* = 20). When the dose of isoflurane increased to a higher level, the FCS of the DMN decreased (Fig. 2e, 1.75% vs QA: *p* > 0.05. One-way ANOVA with Bonferroni's post hoc test, *n* = 20). However, the changes in FCS in different frequency bands were shown to be different with stepwise isoflurane doses. Figure S4 demonstrates that in the lowest isoflurane dose, the FCS of the DMN was increased in the delta band (Figure S4a delta: 0.75% vs QA: *p* < 0.05, one-way ANOVA with Bonferroni's post hoc test, *n* = 20), but decreased in higher frequency bands (Figure S4b-d, theta: 0.75% vs QA: *p* < 0.05, alpha: 0.75% vs QA: *p* < 0.05, and beta: 0.75% vs QA: *p* < 0.05. One-way ANOVA with Bonferroni's post hoc test, *n* = 20). As the isoflurane doses increased, the FCS decreased in the delta band (Figure S4a) and increased in higher frequency bands (Figure S4b-d).

### Time-series fluctuations in DMN dynamic FC analysis

**Fig. 3:**
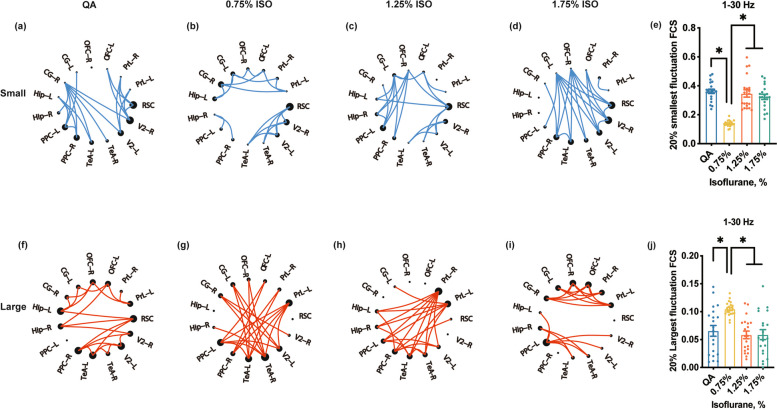
DMN topologies and FCS with the 20% smallest and 20% largest fluctuation FC in the DMN across different states in 1–30 Hz. **a**–**d** Stable network topology (small fluctuation). **e** The FCS of the stable network topology. **f**-**i** Flexible network topology (large fluctuation). **j** The FCS of the flexible network topology. The blue lines indicate the smallest 20% fluctuation of connections based on the fuzzy entropy of PLVs; the red lines indicate the largest 20% fluctuation. The dot size indicates the degree of centrality of the DMN regions. R, right, L, left. *n* = 20

For the QA state, the FC of long-range connectivity between anterior and posterior areas was more stable within 1–30 Hz (Fig. 3a), while the local connectivity was flexible (Fig. 3f). These stable and flexible patterns were broken under the 0.75% isoflurane dose, and adaptively built up with the increase of the isoflurane dose. Specifically, the stable patterns were more local during the 0.75% isoflurane dose, and tended to rebuild the long-range stable connection in 1.25% and 1.75% isoflurane-dose states (Fig. 3b-d). Furthermore, the long-range connections were performed predominantly with flexible patterns under the 0.75% isoflurane dose, and flexible local connections were rebuilt in 1.25% and 1.75% isoflurane doses (Fig. 3g-i). In addition, the FCS of the smallest fluctuation of the DMN showed that the FCS was decreased in the 0.75% isoflurane dose state, and increased in higher isoflurane doses (Fig. 3e, QA vs 0.75%, 1.75% vs 0.75%, 1.25% vs 1.75%, *p* < 0.05, One-way ANOVA with Bonferroni's post hoc test, *n* = 20). However, the FCS of the largest fluctuation of the DMN displayed the opposite trend of variations with isoflurane doses (Fig. 3j, QA vs 0.75%, 1.75% vs 0.75%, 1.25% vs 1.75%, *p* < 0.05, One-way ANOVA with Bonferroni's post hoc test, *n* = 20).

Along with 1–30 Hz, the effect of isoflurane doses on the time-series variation of network connection existed similar tendency in delta bands (Figure S4a, S5a, S6a, S7a). Furthermore, the local connections and long-range connections in theta, alpha, and beta bands were respectively stable and flexible in different doses of isoflurane, which were out of ‘broken up’ and ‘rebuilt up’ (Figure S4b-d, S5b-d, S7b-d, S7f-h).

### The topological features of DMN in different frequency bands

**Fig. 4:**
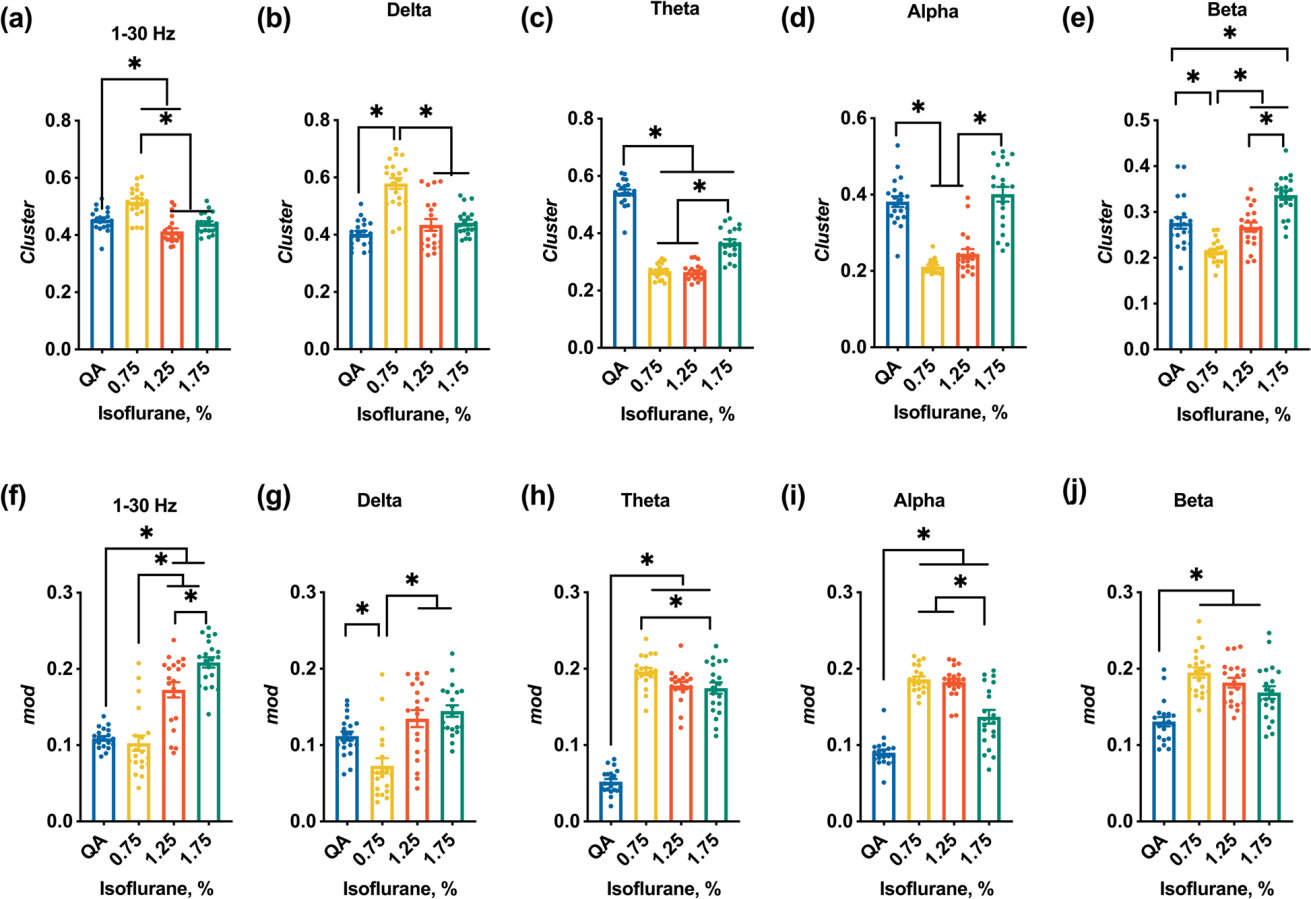
FCS and topological features of the DMN under QA and three isoflurane-dose states. **a**-**e** *Cluster* in the 1–30 Hz and four frequency bands. **f**-**j** *Mod* in the 1–30 Hz and four frequency bands. The dots refer to the *Cluster*, and *Mod* values of each rat. ∗ indicates a significant difference between each state (QA, 0.75% isoflurane, 1.25% isoflurane, and 1.75% isoflurane) using one-way ANOVA with a Bonferroni post hoc test. The level of significance is *p* < 0.05. *n* = 20

Consisting with the FCS, the capability of information processing was increased, which was reflected by the increased* Cluster* (Fig. 4b, *Cluster*: 0.75% vs QA. *p* < 0.01. One-way ANOVA with ﻿Bonferroni’s post hoc test, *n* = 20). In the highest dose of isoflurane, the *Cluster* was decreased (Fig. 4a, *Cluster*: 1.75% vs 0.75%, 1.25% vs 0.75%: *p* < 0.01. One-way ANOVA with ﻿Bonferroni’s post hoc test, *n* = 20). However, the DMN segregation was aggravated with the increase of isoflurane doses (Fig. 4f, *Mod*: 1.25% vs 0.75%, 1.75% vs 0.75%, 1.25% vs 1.75%. *p* < 0.05. One-way ANOVA with ﻿Bonferroni’s post hoc test, *n* = 20).

As for the topological features within DMN shown in Fig. 4, the variation trend of the DMN structure in the delta band is in line with which in 1–30 Hz (Fig. 4b-e), but the trend of variations in the theta, alpha, and beta bands was opposite under isoflurane-dose states (Fig. 5g-j).

### The relationship between stepwise increased doses and DMN

To assess the imposition of stepwise increased isoflurane doses on the DMN in different frequency bands, we entered PSD, FCS, *Mod*, *Cluster*, and FCS with the 20% smallest and 20% largest fluctuation FC into a linear regression model (Stable 3). The results revealed that all these parameters were significantly covaried with isoflurane doses in the theta and beta band. For the 1–30 Hz, delta, and alpha bands, some of the six parameters were also found to vary with isoflurane doses (In detail, for 1–30: FCS, FCS with the 20% smallest FC, *Mod*, and *Cluster*; for delta: FCS with the 20% smallest FC and *Mod*; for alpha: FCS with the 20% smallest and largest FC, PSD, and *Mod*).

## Discussion

To address the alterations of DMN from QA to stepwise increased isoflurane doses administration, we explored the relative power spectrum density, static functional connectivity (FC), the dynamic FC fluctuation measured by fuzzy entropy, overall functional connectivity strength (FCS), *Mod* and *Cluster* for the capability of information processing. The results showed that (1) the adaptive reconstruction patterns of DMN FC existed in spatial and temporal domains under different states; (2) FCS and capability of information processing paradoxically enhanced in 0.75% isoflurane dose compared with QA, which didn’t hint the level of vigilance higher than QA, by contrast, the maintenance of the balance of FC and topological features might play an important role to keep vigilance; (3) the FCS and topological features of holistic DMN have a positive correlation with those in the delta frequency band, which might imply that holistic features of DMN were modulated by slow activity during anesthesia procedure.

### The balance of topological features is broken down by anesthetic

For a healthy brain, topological features with a large *Cluster* and low *Mod* to maintain the balance between global integration and local segregation [8]. Disruption of topological features balance could be found in the disorder of consciousness, and anesthesia [27]. In this study, the increase of *Cluster* could be found in the 0.75% isoflurane-dose state (Fig. 4). With the isoflurane dose increased, this parameter was reversed to decrease and increase, independently. According to ‘﻿the cognitive unbinding theory [36]’, our results hinted that the FC within DMN was stronger, and the capability of the information process was higher than QA in the lowest isoflurane dose. However, this ‘stronger’ and ‘higher’ does not mean the level of vigilance is higher than QA, which might reflect the isoflurane pushes the DMN into a state different from the resting state. In this state, the brain is still positively processing information even though awareness was fading away. With the increase of isoflurane dose, the topology with high *Cluster* was broken down, and the *Mod* in DMN was increased in isoflurane states (Fig. 4), which indicated that DMN has a lower integrative capacity and cannot maintain functional specialization.

### Adaptive reconstruction of DMN FC is isoflurane-dose related

The respiratory rate (RR) and burst-suppression ratio (BSR) (Stable 2), were independently decreased and increased in a dose-related fashion. Consistent with studies about patients [37–40], rodents [41–43], and other species [44–46], indicating that there were deeper physical and cortical suppression changes associated with increasing doses of isoflurane.

In this study, the ‘static’ functional connectivity matrices showed that the DMN in rats under different states (QA and isoflurane-dose states) could be organized in subnetworks, which were identified anterior part, bilateral hippocampus (Hip), and the posterior part by the strength of FC between the brain regions of the DMN. These substructures of DMN generally could be found in rats in sleep [23] and in epilepsy [47]. While the modularized organization is reconstructed in spatial and temporal domains reflected by FC within and among subnetworks under isoflurane-dose states.

Spatially, the degree of modularization and segregation of FC in the anterior and posterior regions were getting higher during the isoflurane states. Even though the Hip-anterior and Hip-posterior FC was irresolute under different states, the anterior–posterior interaction was decreased under isoflurane-dose states. In the temporal domain described by fuzzy entropy, adaptive reconstruction was embodied in anterior–posterior stable connections changed to be more flexible as well as rebuilt along with isoflurane dose increase (Fig. 3b-c, Fig. 3f-g). Our results might suggest that under isoflurane-dose states, network integration and long-range information interaction were disrupted by adaptively reconstructing in DMN. Since ﻿the 'cognitive unbinding theory [36]’ proposed that the anesthetic effects disrupt the ability of effective information transformation across the cortex, our results supported this theory at least in part with DMN.

Surprisingly, along with the increase of isoflurane dose, adaptive reconstruction in time series was the paradoxical rebuilding of long-range stable interactions. We speculate that this ‘rebuilding’ might relate to the ﻿hyperexcitability in the burst-suppression pattern induced by a high dose of anesthetic [14], not relevant to the functional resumption. However, the trends of ‘rebuilding’ were covaried with isoflurane doses. Additionally combined with FCS and topological features under different isoflurane-dose states, these parameters reflect the properties of DMN and were also covaried with anesthetic doses (Fig. 4). These results might suggest that anesthetics might disrupt network optimization and adapt to other optimal constructions in the effect of different isoflurane doses to maintain the brain's intrinsic organization which is expected to prepare for arousal from anesthesia.

### The dose-related response of DMN offers new insights into anesthesia monitoring

The Bispectral Index (BIS) and M-entropy, which have been widely used in clinical settings to monitor the depth of anesthesia in recent decades, their capabilities in predicting emergence from anesthesia, detecting intraoperative awareness, and improving early and intermediate-term survival are limited [6, 48–51]. Our study suggests that the effect of isoflurane on vigilance may be reflected in various aspects of the brain network, such as the spectrum of different frequency bands, network connections and properties, and time-series dynamics of network connections. The results of simple linear-modeling regression demonstrated the *Mod* was significantly affected by isoflurane doses in several frequency bands. The parameters, FCS, 20% smallest FCS, 20% largest FCS, PSD, and *Cluster,* could be detected the significant impose of isoflurane doses in theta, alpha, and beta bands (Stable 3). This discovery could potentially address the limitations of current monitoring methods. Furthermore, a previous study on macaques using resting-state fMRI showed dose-dependent alterations in average connectivity and topological features [52]. Our article demonstrated similar alterations in network features in the electrophysiological DMN which could be detected more easily than fMRI. These dose-dependent alterations in parameters may serve as potential indicators of the level of anesthesia and offer new insights into anesthesia monitoring at the brain network level.

## Limitations and future directions

Experimental limitations should be considered in the current work. First, the GA-induced alterations of unconsciousness are a relative state recruiting local and remote brain regions [7, 53]. These regions distribute from brainstem arousal nuclei to the thalamus and cortex [1]. These anesthetic and arousal core regions are critical for the study of the mechanism of anesthesia. Future work should consider the correlation between these regions and DMN to better understand the effect of isoflurane on the nervous system and the information process mechanism.

Second, our study shows short-term effects of isoflurane exposure, but it is confirmed that alterations still existed in brain tissues after several weeks. Future studies should consider the long-term effects of isoflurane and accumulation confound effects.

Third, because of the shortage of equipment, we could not define the depth of anesthesia and the state of consciousness under each isoflurane dose. Even though the LFP patterns showed distinctive under different isoflurane-dose states, the depth of anesthesia could not be identified by the minimum alveolar concentration (MAC) value.

Fourth, the relationship of network activities in the delta and other frequency bands just stayed at the Pearson correlation, the causality of which should be researched in the future.

Fifth, it is important to note that this study only focused on the effects of isoflurane anesthesia on DMN in rats to reveal the step-increased anesthetic effect. To better understand the potential applications of the dose-related response in the DMN, it would be valuable to compare the effects of distinct anesthetic agents in both animals and human patients.

Sixth, our results demonstrated that alterations in the spectrum, 'static' network connections and properties, and 'dynamic' time-series fluctuations in different frequency bands occurred once the animals were treated with 0.75% isoflurane, potentially indicating the DMN's sensitivity to low-dose anesthetics. Furthermore, the dose-related alterations of these DMN features in different frequency bands reflect the influence of anesthetic doses on brain activities, and suggest that they could be utilized for anesthesia monitoring and optimizing the delivery of anesthetic agents in clinical settings to ensure the safety of GA.

## Conclusion

In conclusion, the present findings indicate that the adaptive reconstruction of DMN is dose-related. These findings might provide a new sight into the mechanism of general anesthesia, pharmacological study, and anesthesia monitoring.

## Supplementary Information

**Additional file1: Figure S1.** Original data QA, Quiet awake state. PrL, prelimbic cortex; OFC, orbitofrontal cortex; Cg, cingulate cortex; RSC, retrosplenial cortex; Hip, hippocampus; PPC, posterior parietal cortex; V2, secondary visual cortex; TeA, auditory/temporal association cortex. R, right, L, left. **Figure S2.** Histological tests were used to determine electrode positions [atlas adapted from (Paxinos and Watson, 2005)]. (a) Electrode position of PrL. (b) Electrode position of OFC. (c) Electrode position of CG. (d) Electrode position of Hip. **STable 2.** Vital parameters. under different isoflurane doses. **Figure S3**. Functional connectivity Functional connectivity under QA and three isoflurane-dose states in different frequency bands. (a-d) Network topology in the delta, theta, alpha, and beta bands in QA state, 0.75%, 1.25%, and 1.75%. PrL, prelimbic cortex; OFC, orbital cortex; Cg, cingulate cortex; Hip, hippocampus; PPC, posterior parietal cortex; TeA, auditory/temporal association cortex; V2, secondary visual cortex; RSC, retrosplenial cortex. *n* = 20. **Figure S4. **FCS of the DMN under QA and three isoflurane-dose states in the four frequency bands. The dots refer to the FCS values of each rat. ∗ indicates a significant difference between each state (QA, 0.75% isoflurane, 1.25% isoflurane, and 1.75% isoflurane) using one-way ANOVA with a Bonferroni *post hoc* test. The level of significance is *p* < 0.05. n = 20. **Figure S5.** DMN topologies with the 20% smallest fluctuation FC in the DMN across different states are measured by fuzzy entropy in 1-30 Hz in different bands. (a–d) 20% of smallest the connections are based on fuzzy entropy of network topology in the delta, theta, alpha, and beta bands across QA, 0.75%, 1.25%, and 1.75% isoflurane-dose states. The blue lines indicate the top 20% largest variation of connections based on the fuzzy entropy of PLVs. The dot size indicates the degree of centrality of the DMN regions. *n* = 20. **Figure S6.** DMN topologies with the 20% largest fluctuation FC in the DMN across different states are measured by fuzzy entropy in 1-30 Hz. (a–d) 20% of the largest variations of the connections are based on fuzzy entropy of network topology in the delta and beta bands across QA, 0.75%, 1.25%, and 0.75% states. The red lines indicate the smallest 20% of connections based on the fuzzy entropy of PLVs. *n* = 20. **Figure S7.** 20% smallest and 20% largest fluctuation FC in the DMN across different states in the four frequency bands. (a–d) The FCS of the stable network topology. (e-h) The FCS of flexible network topology (large fluctuation). (a-d) The FCS of the flexible network topology. *n* = 20. **STable 3. **Simple linear regression between different isoflurane doses and FCS, PSD, and topological features.

## Data Availability

All data generated or analysed during this study are included in this published article and data will be made available from the corresponding author on reasonable request.
