# Supplementary material for: Dose-related adaptive reconstruction of DMN in isoflurane administration: a study in the rat
Source: BMC Anesthesiol. 2023 Jun 28;23:224. doi: 10.1186/s12871-023-02153-6 (PMC10303294; doi:10.1186/s12871-023-02153-6)
Supplement: Supplementary file 1 — Additional file1: Figure S1. Original data QA, Quiet awake state. PrL, prelimbic cortex; OFC, orbitofrontal cortex; Cg, cingulate cortex; RSC, retrosplenial cortex; Hip, hippocampus; PPC, posterior parietal cortex; V2, secondary visual cortex; TeA, auditory/temporal association cortex. R, right, L, left. Figure S2. Histological tests were used to determine electrode positions [atlas adapted from (Paxinos and Watson, 2005)]. (a) Electrode position of PrL. (b) Electrode position of OFC. (c) Electrode position of CG. (d) Electrode position of Hip. STable 2. Vital parameters. under different isoflurane doses. Figure S3. Functional connectivity Functional connectivity under QA and three isoflurane-dose states in different frequency bands. (a-d) Network topology in the delta, theta, alpha, and beta bands in QA state, 0.75%, 1.25%, and 1.75%. PrL, prelimbic cortex; OFC, orbital cortex; Cg, cingulate cortex; Hip, hippocampus; PPC, posterior parietal cortex; TeA, auditory/temporal association cortex; V2, secondary visual cortex; RSC, retrosplenial cortex. n = 20. Figure S4. FCS of the DMN under QA and three isoflurane-dose states in the four frequency bands. The dots refer to the FCS values of each rat. ∗ indicates a significant difference between each state (QA, 0.75% isoflurane, 1.25% isoflurane, and 1.75% isoflurane) using one-way ANOVA with a Bonferroni post hoc test. The level of significance is p < 0.05. n = 20. Figure S5. DMN topologies with the 20% smallest fluctuation FC in the DMN across different states are measured by fuzzy entropy in 1-30 Hz in different bands. (a–d) 20% of smallest the connections are based on fuzzy entropy of network topology in the delta, theta, alpha, and beta bands across QA, 0.75%, 1.25%, and 1.75% isoflurane-dose states. The blue lines indicate the top 20% largest variation of connections based on the fuzzy entropy of PLVs. The dot size indicates the degree of centrality of the DMN regions. n = 20. Figure S6. DMN topologies with the 20% l [file 12871_2023_2153_MOESM1_ESM.docx]

**Supplementary materials**


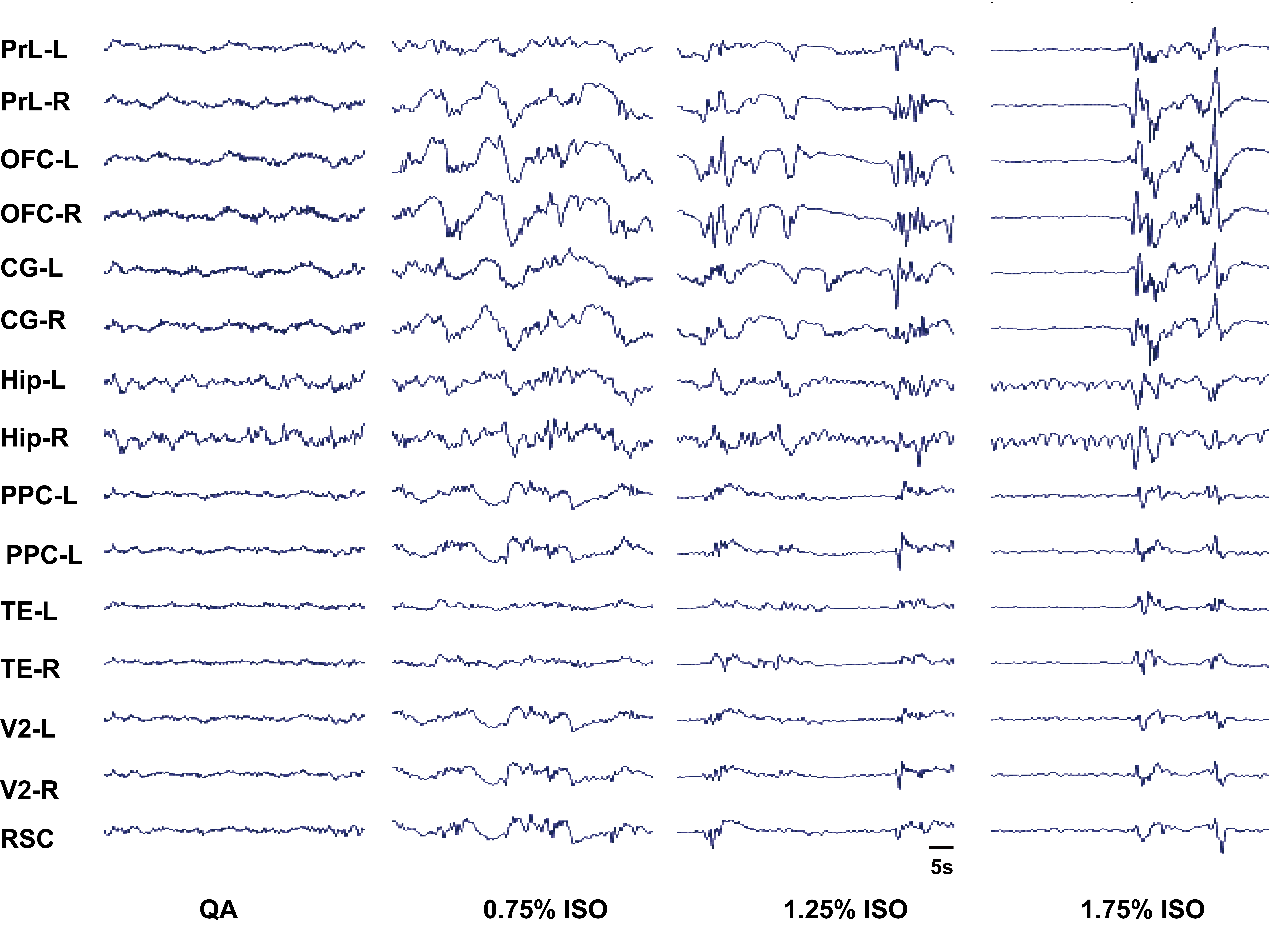


**Figure S1. Original data** QA, Quiet awake state. PrL, prelimbic cortex; OFC, orbitofrontal cortex; Cg, cingulate cortex; RSC, retrosplenial cortex; Hip, hippocampus; PPC, posterior parietal cortex; V2, secondary visual cortex; TeA, auditory/temporal association cortex. R, right, L, left

**Histology examination**

After the experiment, all animals were anesthetized with sodium pentobarbital (15 mg/kg) and intracardially perfused with phosphate-buffered solution (PBS) followed by 4% paraformaldehyde (PFA)/PBS. The brains were removed and fixed in PFA/PBS for 4 hours and then moved to 30% sucrose diluted in PFA/PBS. After dehydration, 45 $\mu$m coronal sections were prepared with a freezing microtome (Leica CM1950; Leica Biosystems Nussloch GmbH Inc., Heidelberger, Germany). The brain sections were stained with ferric chloride solution on poly-L-lysine-coated slides, cover-slipped with DPX mountant, and digitally photographed with a BX53 microscope (Olympus, Tokyo, Japan). Twenty rats were included in the analyses based on the standard of the correct location of electrode tips in all DMN regions (S2).


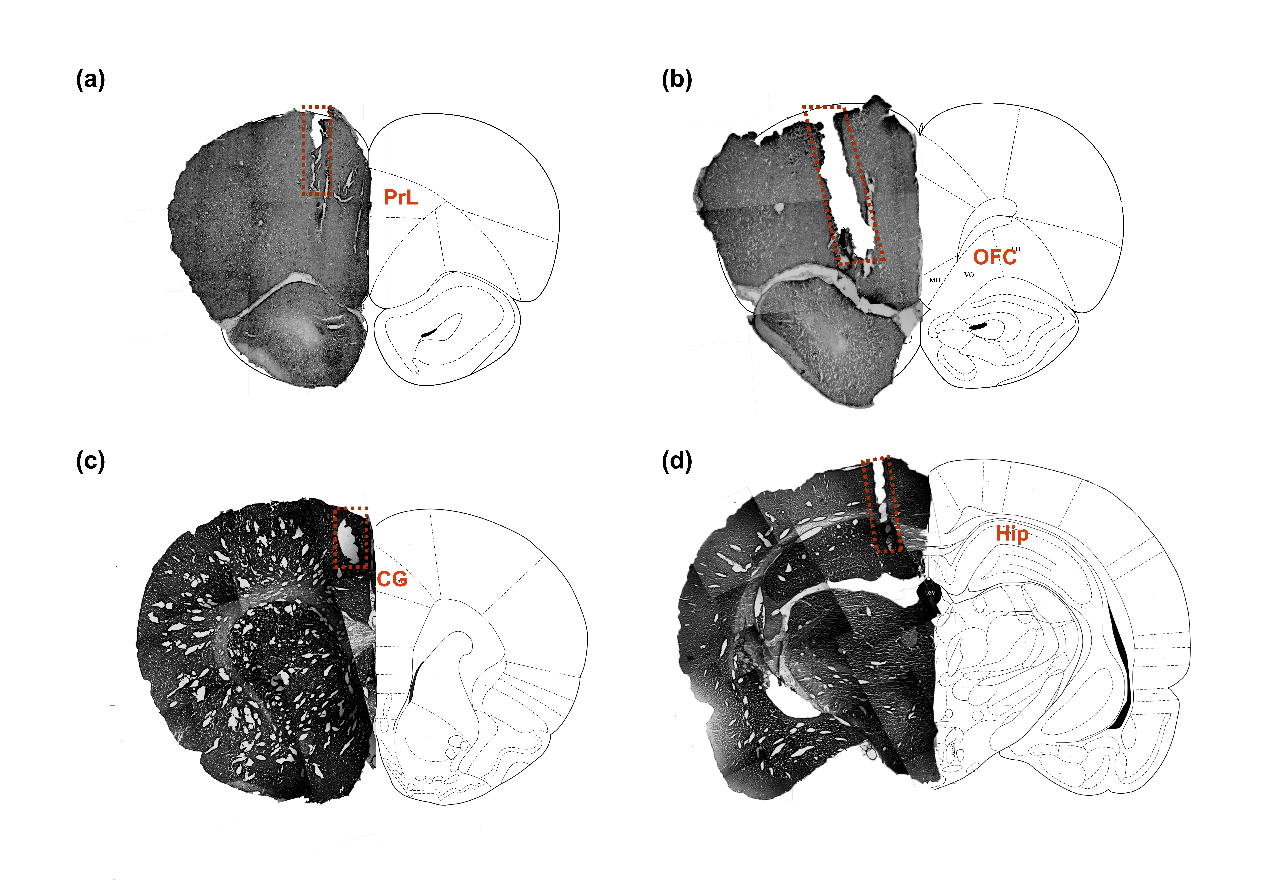


**Figure S2. Histological tests were used to determine electrode positions** [atlas adapted from (Paxinos and Watson, 2005)]. (a) Electrode position of PrL. (b) Electrode position of OFC. (c) Electrode position of CG. (d) Electrode position of Hip.

**Computation of the burst suppression ratio**

To estimate the effect of isoflurane on the nervous system, we quantified the burst suppression ratio (BSR). BSR was defined as the fraction of isoelectric periods occurring per epoch. A suppression period was defined as a period that started with LFP amplitudes that remained isoelectric for more than 0.5 s. Ten minutes of data from each isoflurane dose and quiet-awake (QA) were selected for each epoch (Choi et al., 2016; Detsch, Schneider, Kochs, Hapfelmeier, & Werner, 2000).

$$\begin{aligned} BSR \left( \% \right)=\frac{total time of suppression}{epoch length}\times100\%\#\left( 1 \right) \end{aligned}$$

**STable 2. Vital parameters under different isoflurane doses.**

| Isoflurane  concentration (%) | Vital parameters | | | | |
| --- | --- | --- | --- | --- | --- |
|  | RFS (%) | M (%) | RR (times/min) | | BSR (%) |
| 0 | 100 | 100 | 123$\pm$4 | | 0 |
| 0.75 | 75 | 0 | 81$\pm$3$*$ | | $7.4\pm$3.4 |
| 1.25 | 0 | 0 | 61$\pm$3$*\dagger$ | | 22$\pm$4.5∗$\dagger$ |
| 1.75 | 0 | 0 | 44$\pm$4*$\dagger\Delta$ | 40.7$\pm5.8*\dagger\Delta$ | |
| *p* |  |  | <0.001 | | <0.001 |

Data are the mean ± standard error of the mean. ∗ Significantly (*p* < 0.05) different from the respective value during the control (quiet-awake state) experiments. † Significantly (*p* < 0.05) different from the respective value during the 0.75% isoflurane dose. ∆ Significantly (p<0.05) different from the 1.25% isoflurane. The *p* value refers to significant overall changes (Brown-Forsythe and Welch ANOVA tests, *post hoc* Dunnett’s T3 tests). RFS, response to forepaw stimuli; M, movements; RR, respiratory rate; BSR, burst suppression ratio. n = 20.


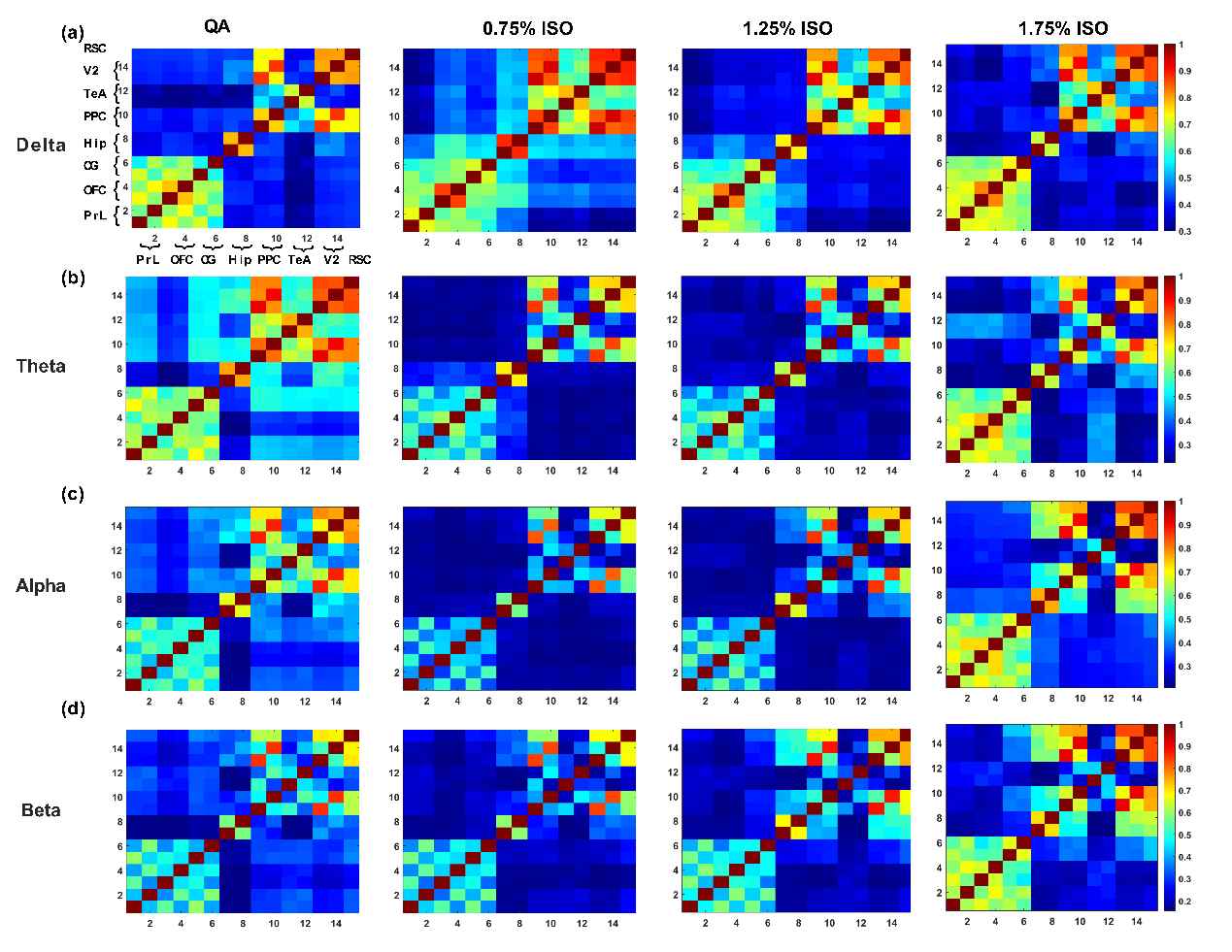


**Figure S3. Functional connectivity Functional connectivity under QA and three isoflurane-dose states in different frequency bands.** (a-d) Network topology in the delta, theta, alpha, and beta bands in QA state, 0.75%, 1.25%, and 1.75%. PrL, prelimbic cortex; OFC, orbital cortex; Cg, cingulate cortex; Hip, hippocampus; PPC, posterior parietal cortex; TeA, auditory/temporal association cortex; V2, secondary visual cortex; RSC, retrosplenial cortex. n = 20.


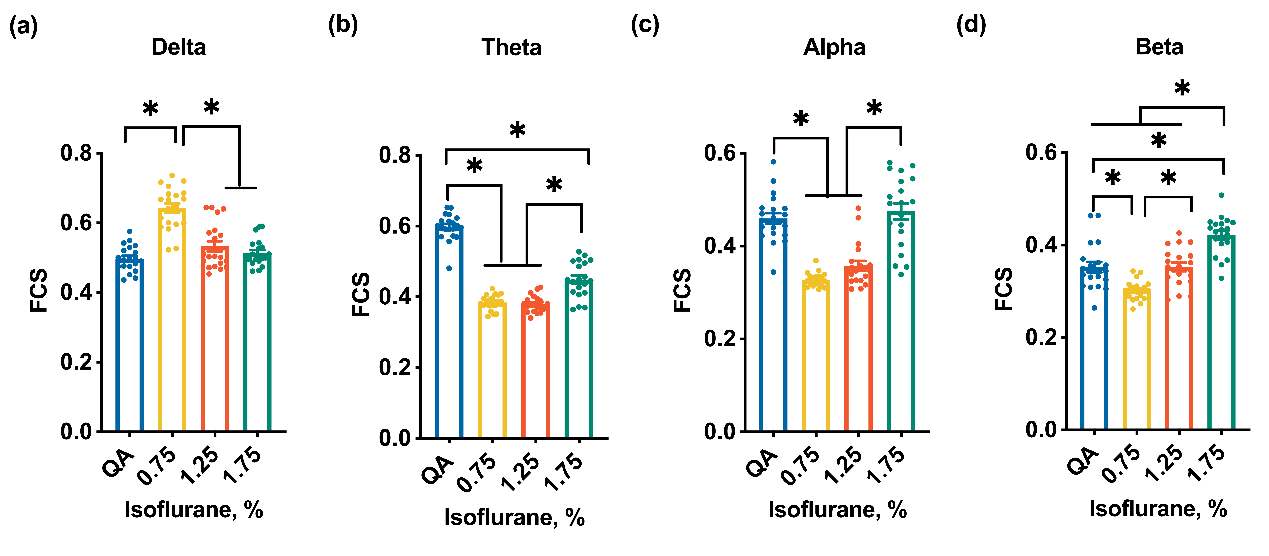


**Figure S4 FCS of the DMN under QA and three isoflurane-dose states in the four frequency bands.** The dots refer to the FCS values of each rat. ∗ indicates a significant difference between each state (QA, 0.75% isoflurane, 1.25% isoflurane, and 1.75% isoflurane) using one-way ANOVA with a Bonferroni *post hoc* test. The level of significance is *p* < 0.05. n = 20.


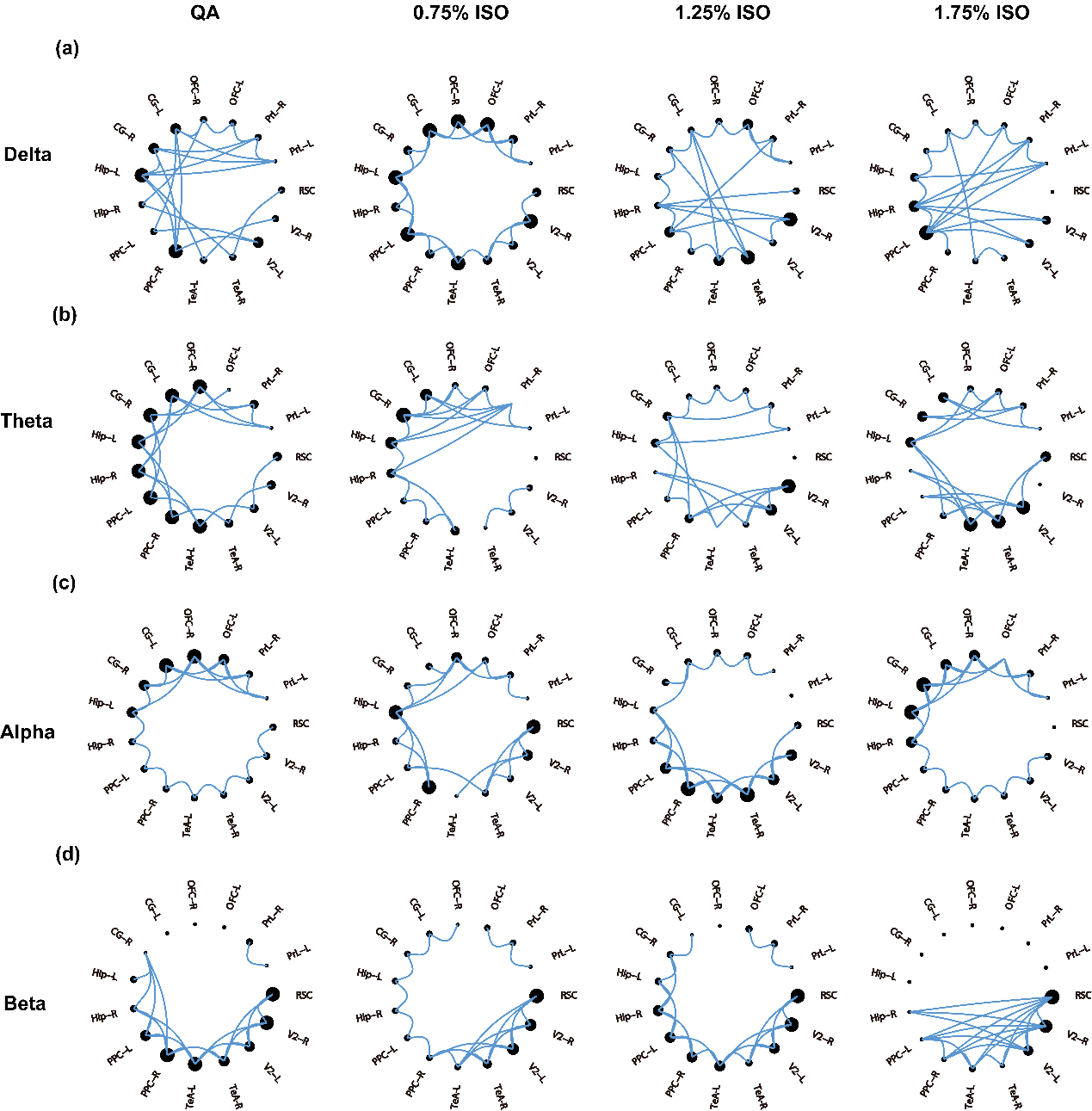


**Figure S5.** **DMN topologies with the 20% smallest fluctuation FC in the DMN across different states are measured by fuzzy entropy in 1-30 Hz in different bands.** (a–d) 20% of smallest the connections are based on fuzzy entropy of network topology in the delta, theta, alpha, and beta bands across QA, 0.75%, 1.25%, and 1.75% isoflurane-dose states. The blue lines indicate the top 20% largest variation of connections based on the fuzzy entropy of PLVs. The dot size indicates the degree of centrality of the DMN regions. n = 20.


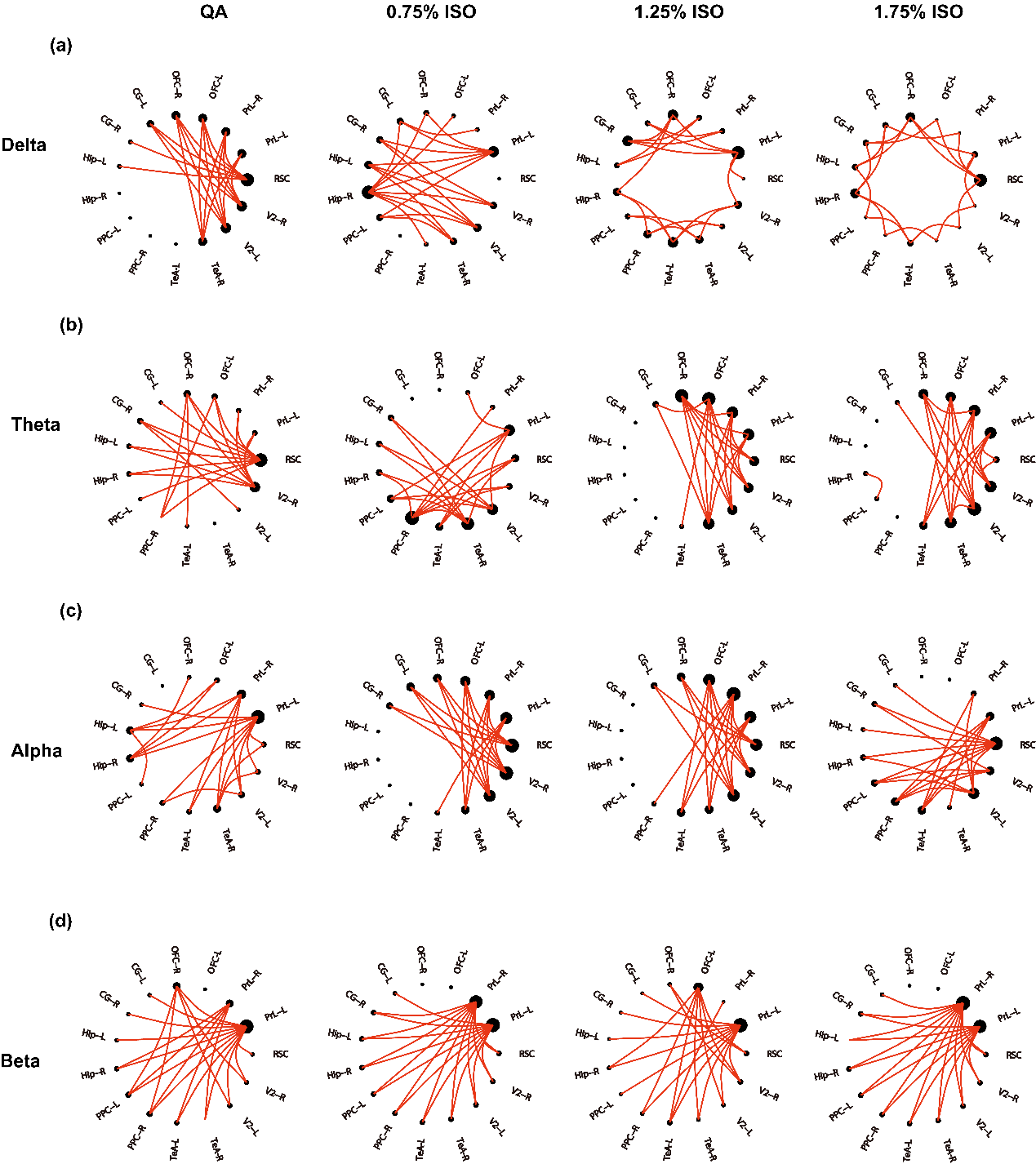


**Figure S6. DMN topologies with the 20% largest fluctuation FC in the DMN across different states are measured by fuzzy entropy in 1-30 Hz.** (a–d) 20% of the largest variations of the connections are based on fuzzy entropy of network topology in the delta and beta bands across QA, 0.75%, 1.25%, and 0.75% states. The red lines indicate the smallest 20% of connections based on the fuzzy entropy of PLVs. n = 20.


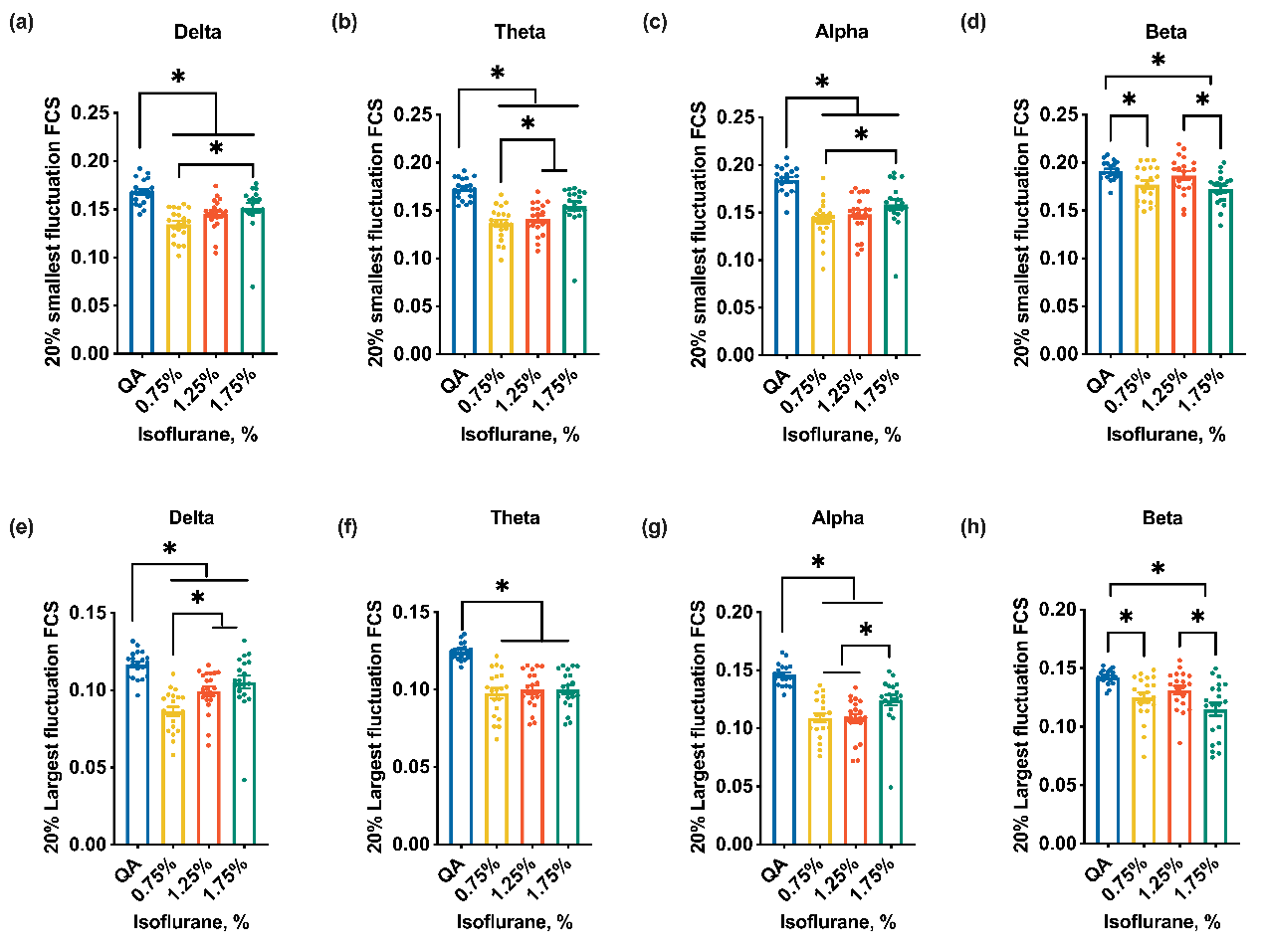


**Figure S7** **20% smallest and 20% largest fluctuation FC in the DMN across different states in the four frequency bands**. (a–d) The FCS of the stable network topology. (e-h) The FCS of flexible network topology (large fluctuation). (a-d) The FCS of the flexible network topology. n = 20.

|  |  | 1-30 Hz | | Delta | | Theta | | Alpha | | Beta | |
| --- | --- | --- | --- | --- | --- | --- | --- | --- | --- | --- | --- |
|  |  | R^2^ | Sig | R^2^ | Sig | R^2^ | Sig | R^2^ | Sig | R^2^ | Sig |
| FCS | Isoflurane dosages | **-3.238** | **.002** | -.156 | .907 | **-8.893** | **.000** | .322 | .822 | **3.947** | **.000** |
| 20% Smallest FCS | Isoflurane dosages | .336 | .871 | **-.853** | **.017** | **-1.062** | **.004** | **-1.460** | **.001** | **-.864** | **.005** |
| 20% Largest FCS | Isoflurane dosages | -.460 | .642 | -.530 | .081 | **-1.341** | **.000** | **-1.344** | **.000** | **-1.349** | **.000** |
| PSD | Isoflurane dosages | .694 | .353 | -4.669 | .055 | **-3.314** | **.007** | **5.306** | **.000** | **2.676** | **.000** |
| *Mod* | Isoflurane dosages | **.492** | **.000** | **0.168** | **.000** | **0.490** | **.000** | **0.168** | **.000** | **0.122** | **.002** |
| *Cluster* | Isoflurane dosages | **0.056** | **0.034** | 0.001 | 0.776 | **0.331** | **.000** | 0.000 | 0.8716 | **0.135** | **.001** |

**STable 3 Simple linear regression between different isoflurane doses and FCS, PSD, and topological features**
